# Potential Therapeutic Role of Pituitary Adenylate Cyclase-Activating Polypeptide for Dry Eye Disease

**DOI:** 10.3390/ijms23020664

**Published:** 2022-01-08

**Authors:** Takahiro Hirabayashi, Junko Shibato, Ai Kimura, Michio Yamashita, Fumiko Takenoya, Seiji Shioda

**Affiliations:** 1 Clinical Medicine Research Laboratory, Shonan University of Medical Sciences, 16-48, Kamishinano, Totsuka-ku, Yokohama 244-0806, Japan; t-hirabayashi@hoshi.ac.jp (T.H.); j-shibatou@hoshi.ac.jp (J.S.); a-kimura@hoshi.ac.jp (A.K.); 2 Department of Physiology and Molecular Sciences, School of Pharmacy, Hoshi University, 2-4-41 Ebara, Shinagawa-ku, Tokyo 142-8501, Japan; d1902@hoshi.ac.jp (M.Y.); kuki@hoshi.ac.jp (F.T.); 3 Department of Clinical Pharmacy, Faculty of Pharmaceutical Sciences, Shonan University of Medical Sciences, 16-48, Kamishinano, Totsuka-ku, Yokohama 244-0806, Japan

**Keywords:** dry eye disease, medication, drug discovery, PACAP, PAC1-R, tear secretion, peptide therapeutics

## Abstract

Dry eye disease (DED) is caused by a reduction in the volume or quality of tears. The prevalence of DED is estimated to be 100 million in the developed world. As aging is a risk factor for DED, the prevalence of DED is expected to grow at a rapid pace in aging populations, thus creating an increased need for new therapies. This review summarizes DED medications currently in clinical use. Most current medications for DED focus on stimulating tear secretion, mucin secretion, or suppressing inflammation, rather than simply replenishing the ocular surface with moisture to improve symptoms. We recently reported that the neuropeptide PACAP (pituitary adenylate cyclase-activating polypeptide) induces tear secretion and suppresses corneal injury caused by a reduction in tears. Moreover, it has been reported that a PACAP in water and a 0.9% saline solution at +4 °C showed high stability and achieved 80–90% effectiveness after 2 weeks of treatment. These results reveal PACAP as a candidate DED medication. Further research on the clinical applications of PACAP in DED is necessary.

## 1. Introduction

Dry eye disease is an umbrella term covering several symptoms associated with compromised ocular lubrication, or in other words a reduced quantity of tears on the ocular surface. Dry eye disease (DED) or dysfunctional tear syndrome (DTS), clinically referred to as keratoconjunctivitis sicca, is defined by the Tear Film and Ocular Surface Society’s Dry Eye Workshop II (TFOS DEWS II) panel as “a multifactorial disease of the ocular surface characterized by a loss of homeostasis of the tear film and accompanied by ocular symptoms, such as discomfort and visual disturbance” [1]. DED is a common disorder of the ocular surface that affects millions of people worldwide, with varying severity. In many patients, the condition is chronic and requires long-term treatment. As the population ages, the prevalence of dry eye is likely to increase, yet the condition is often underrecognized and undertreated. This review describes the current knowledge of the causes and symptoms of DED along with current therapies. An overview of studies on the use of PACAP as a candidate drug for DED is provided.

## 2. Dry Eye Disease—Signs, Symptoms, and Causes

The DED can be divided into two major categories: the aqueous tear-deficient type and the evaporative type [2]. Aqueous tear-deficient type DED is characterized by a decreased secretion of tears from the lacrimal glands, whereas evaporative type DED results from increased evaporation of tear fluid from the eye surface. These conditions are not mutually exclusive; in fact, they often overlap. Both types of DED can be accompanied by ocular surface inflammation and high or unstable tear osmolarity. Around 10% of patients with dry eye have a solely aqueous tear-deficient type DED. Hyperevaporative type DED, mostly caused by dysfunction of the meibomian glands, and a mixed aqueous tear-deficient type/hyperevaporative type account for more than 80% of cases [3].

The most common signs and symptoms of DED include eye fatigue, blurred eyes, double vision, sore eyes, a stinging or burning sensation, eye inflammation, and focusing problems [4]. Most DED patients experience mild inflammation with no long-term effects. DED can lead to tiny abrasions on the ocular surface. If the condition is left untreated, it can produce complications that can cause eye damage, resulting in impaired vision or even vision loss. The burden of DED on affected patients is significant, with effects on visual function, living and work activities, and quality of life [5]. A reduced quality of life in daily activities was reported by 60% of DED patients, while 38% of DED patients complained of reduced efficiency at work [6,7]. Direct costs from doctor’s consultation fees, medications, and treatments, and indirect costs from a reduced quality of life make DED a substantial economic burden in addition to its clinical burden [5].

Various studies have reported a relatively wide range of prevalence estimates, ranging from 7 to 33% [8] and amounting to as many as 20 million people in the USA and 100 million in the developed world [9]. The variability of symptoms has contributed to a wide variation in reported prevalence figures. The prevalence of DED is higher among women as compared to men and increases with age [5]; among older people the condition affects up to 70% [10]. Women have an increased prevalence of DED after menopause, and treatment with estrogen can worsen symptoms [11]. On the contrary, androgen treatment improves DED symptoms [12]. About two-thirds of contact lens prescriptions are for women, and women are more likely than men to undergo refractive surgery such as LASIK (laser in situ keratomileusis) [13]. These factors are associated with DED, in addition to which the number of patients diagnosed with the condition has increased in recent years, which could be due to the wearing of contact lenses or the popularity of video display devices [14,15].

Many medications have been reported to trigger DED, including diuretic agents, β-blockers, other antihypertensive agents, antihistamines, antidepressant agents, anxiolytic agents, anticonvulsant agents, antipsychotic agents, and antispasmodic agents [16]. Clinical conditions that increase the risk of DED include autoimmune diseases, such as rheumatoid arthritis, sarcoidosis, and Sjögren’s syndrome, and chronic conditions such as thyroid abnormalities, diabetes, and hepatitis C infection [17]. Furthermore, DED is significantly associated with anxiety disorders and depression. One large population-based cross-sectional study found manifest depression in 13.7% of patients with dry eye disease, compared with 8.6% in the control group [6,18].

DED has many causes, which often overlap and interact. Its putative pathogenetic mechanisms include hyperosmolarity of the tear film and inflammation of the ocular surface and lacrimal gland. The most common cause of DED is increased evaporation of the tear film, typically as a result of Meibomian gland dysfunction, which manifests as plugged gland orifices, a perturbed lipid layer in the tear film, and inflammation of the lid margin. An insufficient lipid component may lead to a more rapid evaporation of the aqueous tear component.

Many cellular and molecular components contribute to the pathogenesis of DED. The reduced tear secretion that is characteristic of aqueous-deficient type DED results in tear film hyperosmolarity associated with an inflammatory cascade that produces various proinflammatory cytokines, such as interleukin-1α (IL-1α), IL-1β, tumor necrosis factor- α (TNF-α), and matrix metalloproteinase 9 [19].

## 3. Common DED Medications

Given the pathogenesis of dry eye disease, achieving tear film stability is crucial, i.e., the qualitative and quantitative improvement of tears. The basic strategies include increasing the amount of liquid on the ocular surface, decreasing tear evaporation, and augmenting the lipid content or lubricity of the tears. These strategies can be used in the treatment of aqueous-deficient type DED or evaporative type DED and are aimed at increasing tear volume or improving the quality of the tear film.

### 3.1. Artificial Tears

Tear components are secreted from the main lacrimal gland, accessory lacrimal gland (Krause’s and Wolfring’s glands), meibomian gland, and the corneal and conjunctival epithelia. Human tears, which comprise water, proteins, electrolytes, and lipids, function to keep the ocular surface moist and protect the cornea from trauma and infection. Tear replenishment is one of the principal approaches for the treatment of DED [20,21] with artificial tears (ATs), the mainstay of therapy for DED of all severity grades. ATs are thought to improve DED symptoms by mimicking tears and improving tear stability and properties including osmolarity [22]. Medications termed ATs and which contain carboxymethyl cellulose (CMC), hydroxypropyl methyl cellulose (HPMC), polyvinyl alcohol, polyvinylpyrrolidone, or polyethylene glycol, are available [21]. CMC is a polysaccharide with mucoadhesive properties and has been shown to bind to corneal epithelial cells. HPMC is also a polysaccharide polymer produced from cellulose and induces moistening and lubrication of the eye surface. Osmoprotectants, such as trehalose and erythritol, are components that protect cells against hyperosmolar stress-mediated injury. Since hyperosmolarity is one the symptoms of DED, these are emerging ingredients of artificial tears [23].

The toxic effects of preservatives in topical ocular medications, including benzalkonium chloride, can lead to inflammation of the conjunctiva and tear film instability, thereby causing or exacerbating the symptoms and signs of dry eye. For this reason, in DED patients requiring frequent treatment with ATs, preservative-free formulations may provide better efficacy [24].

In 2018, ATs accounted for USD 2.2 billion in global sales, and their market continues to grow [23]. However, although ATs provide only temporary symptomatic relief, they do not address the underlying pathophysiology of DED, and the outcome is not always satisfactory [25]. Some AT-like medications also include eyedrops prepared with sterile, saline-diluted serum derived from the patient’s blood (autologous serum eye drops) for severe cases of DED [26]. Autologous serum eye drops contain a multitude of epitheliotropic growth factors and anti-inflammatory substances. Nevertheless, evidence from examined trials showed that while autologous serum eye drops may have a small short-term benefit when compared to ATs, there is no evidence of improvement after 2 weeks [27].

### 3.2. Hyaluronic Acid Ophthalmic Solution

ATs may be used in combination with other biologically active ingredients.

Hyaluronic acid (HA) is a glycosaminoglycan disaccharide made of β-D (1→3) glucuronic acid and β-D (1→4) N-acetyl-β-D-glucosamine; it is present in connective tissue, synovial fluid, and in the aqueous humor, and is vitreous. HA is thought to stabilize the tear film by promoting water retention because of its three-dimensional structure, in addition to water supplementation.

CD44 (cluster of differentiation 44), a transmembrane cell surface adhesion molecule, is a receptor for HA. CD44 is a ubiquitously present glycoprotein on the surface of mammalian cells, including retinal Müller, pericytes, retinal pigment epithelial, corneal epithelial, and ganglion cells [28]. The interaction between CD44 and HA plays a regulatory role in cell physiology. HA promotes corneal epithelial wound healing in animal experiments [29]. It has been suggested that HA promotes cell migration and corneal wound healing by interacting with CD44 [30].

The first ophthalmic medical device containing HA was approved by the Food and Drug Administration (FDA) in the 1980s [31]. In Japan and other countries, HA eye drops have been used for many years to treat DED in combination with preservative-free artificial ophthalmic solutions under the product name Hyalein^®^.

### 3.3. Loteprednol Etabonate Ophthalmic Solution

Inflammation is one of the major symptoms of DED. Corticosteroids are potent anti-inflammatory agents that have been used to treat ocular inflammation. Topical corticosteroids are an effective treatment option for moderate to severe DED with ocular inflammation.

Although corticosteroids have proven short-term efficacy, their long-term use may cause side effects such as cataract formation, the induction of glaucoma and intraocular pressure [32].

Loteprednol etabonate (LE) is an ocular corticosteroid with improved safety compared with other corticosteroids. LE was synthesized from the modification of an inactive metabolite of prednisolone acetate [33]. LE undergoes deesterification of an inactive carboxylic acid metabolite after exerting its effect, thus reducing the potential for adverse reactions [34].

There has been a trend in the use of LE in combination with cyclosporin A (CsA), described below, for the treatment of dry eye in clinical practice. LE is marketed by Bausch and Lomb under the names Lotemax^®^ and Loterex^®^.

### 3.4. Cyclosporin A Ophthalmic Solution

Corticosteroids are widely used for treating the symptoms of ocular inflammation. Unfortunately, their long-term use is associated with serious side effects such as glaucoma and cataract, as described above. Nonsteroidal anti-inflammatory compounds are therefore preferred for the long-term treatment of DED patients, and CsA has demonstrated positive results in an animal model of DED [35] and in clinical trials [36].

CsA, which was isolated from the fungus *Tolypocladium inflatum*, is a cyclic peptide of 11 amino acids; it contains a single D-amino acid, which is rarely encountered in nature [37].

CsA inhibits the calcineurin–phosphatase pathway by complex formation with cyclophilin and thus the inhibition of the production of cytokines such as IL-2 involved in the regulation of T-cell activation. Based on these properties, CsA becomes the immunosuppressive drug in organ transplantation [38].

The effects of CsA on DED are the inhibition of T-cell activation and reduction in the cyclophilin-mediated gene transcription of IL-2 and IL-6 [39]. Furthermore, CsA inhibits caspase activation, and CsA protects human conjunctival epithelial cells via its anti-apoptotic effect and improves corneal surface conditions [40]. Moreover, topical application of CsA leads to increased tear fluid production, possibly via the local release of parasympathetic neurotransmitters [38].

The side effects of CsA were reported to include feelings of burning and itching, an altered sensation of taste, blurred vision, and discharge [41]. The long-term use of CsA at high doses is associated with an increased risk of cancer [42]. Unlike ATs, CsA must be administered for a period of up to several months to achieve therapeutic effects for DED. In the United States, a CsA ophthalmic solution (Restasis^®^) was approved in 2003 based on its ability to increase tear production in DED patients. Cheaper generic alternatives are available in some countries.

### 3.5. Rebamipide Ophthalmic Solution

Mucins are heavy molecular glycoproteins formed by numerous sugar chains linked to a core protein called apomucin. In mucins, N-acetylgalactosamine is linked to serine or threonine, which are two of the amino acids of the protein core, in what is called an O-type glycosylation [43].

Mucins are produced by conjunctival goblet cells and released from the corneal and conjunctival epithelium. In healthy eyes, mucins provide an even, slippery tear coating, minimizing friction and protecting the cornea during blinking. Mucins play an important role in the lubrication and defense of the ocular surface [44].

It is considered that disorders in keratoconjunctival mucin coverage inhibit the uniform extension of tears over the ocular surface, producing a tear film with an uneven thickness and an aggravated ocular surface environment [45].

Because the expression of both secreted and membrane-associated mucins is decreased in DED, those functions are disrupted on the ocular surface [46]. Improving the mucin coating on the ocular surface in DED patients is considered very important for dry eye treatment. A decrease in mucin secretion in DED highlights the need for medications that overcome this problem.

Rebamipide is an amino acid analog of 2(1H)-quinolinone, and its major physiological properties are the stimulation of prostaglandin and mucus glycoprotein synthesis [47,48]. Due to these actions, rebamipide (Mucosta^®^) was marketed in 1990 as an oral therapeutic drug to treat gastric mucosal disorders and gastritis (Mucosta^®^). Subsequent investigations showed that rebamipide increases mucin-like substances and improved corneal/conjunctival injury. Based on these results, rebamipide is currently marketed in Japan, the People’s Republic of China, Indonesia, Malaysia, and Thailand to treat DED.

Further research on the effect of rebamipide on conjunctival goblet cell counts found that a rebamipide ophthalmic suspension increased numbers of these cells in normal rabbits [49]. Moreover, rebamipide increases tear stability by activating epithelial differentiation as opposed to increasing the tear volume [50]. The most frequently observed adverse event for rebamipide ophthalmic suspension in a phase III clinical study was dysgeusia (bitter taste) [51].

### 3.6. Diquafosol Ophthalmic Solution

Diquafosol is a stable derivative of uridine 5′-triphosphate, a purinoreceptor P2Y2 receptor agonist. The P2Y2 receptor is widely distributed throughout the body and is expressed in the conjunctival epithelium, including goblet cells in the eye [52,53]. P2Y2 receptor agonists act on P2Y2 receptors in cell membranes and activate phospholipase C via G proteins to produce inositol triphosphate. Thus, calcium ion (Ca^2+^) release is induced from the cell endoplasmic reticulum, which elevates intracellular Ca^2+^ concentrations and induces various physiological responses [52]. Through the P2Y2 receptor, diquafosol facilitates fluid transport from the serosal to the mucosal side via chloride channel activation following elevation of the Ca^2+^ concentration in epithelial cells on the ocular surface. Diquafosol also induces fluid secretion from conjunctival epithelial cells and mucin secretion from conjunctival goblet cells directly on the ocular surface [54]. This drug also upregulates the expression of mucin genes in corneal epithelial cells [55].

Diquafosol is marketed under the product name Diquas^®^, which was launched in Japan at the end of 2010 as a drug for the treatment of dry eye with a novel mechanism of action involving the stimulation of tear and mucin secretion. Diquas^®^ has also been launched in other Asian countries such as China, Korea, Thailand, and Vietnam. Diquafosol ophthalmic solution as a drug for dry eye treatment is expected to have therapeutic effects on disease symptoms that do not respond sufficiently to standard treatments. For example, an option for adding 3% diquafosol ophthalmic solution in combination with 0.1% HA ophthalmic solution has been suggested for DED where HA monotherapy is insufficient [56].

Diquafosol promotes corneal epithelial wound healing through P2Y2 receptor activation via the mitogen-activated protein kinase (MAPK) cascade. Diquafosol increased the levels of phospho-Erk1/2, phospho-90RSK, phospho-Akt, and IκB-α in human corneal epithelial cells, whereas it significantly decreased the levels of NF-κB-p65, IL-1β, and TNF-α in these cells. On the other hand, scopolamine hydrobromide-induced apoptosis was enhanced in a DED rat model. This response was markedly reduced, and the level of phosphorylated p90RSK and phosphorylated ERK1/2 were upregulated and IL-1βdownregulated by diquafosol [57]. P2Y2 receptors are also known to be present in the meibomian glands, suggesting that diquafosol could be effective against dry eye caused by Meibomian gland dysfunction [52].

The observed adverse drug reactions for diquafosol ophthalmic solutions in a clinical study were irritation, eye discharge, conjunctival injection, eye pain, eye pruritus, foreign body sensation, and eye discomfort [55]. Although rebamipide ophthalmic solution and diquafosol ophthalmic solution have similar effects on DED, further studies are needed to clarify their differences.

### 3.7. Lifitegrast Ophthalmic Solution

Integrins are cell-surface proteins expressed on a variety of cells. They are upregulated in response to pathological insult and also play important roles in integrating signals among various cells. The integrin LFA-1 (lymphocyte function-associated antigen 1) is a leukocyte cell-surface glycoprotein and a modulator of T cell activation and proliferation [58]. ICAM-1, also known as CD54, is a cell-surface glycoprotein member of the immunoglobulin (Ig) superfamily and consists of five extracellular Ig-like domains, a transmembrane domain, and a short cytoplasmic domain [59]. ICAM-1 initiates T-cell activation and can trigger T-cell migration through binding to LFA-1. ICAM-1 has two isoforms consisting of membrane-bound and soluble ICAM-1 and is constitutively expressed at low levels on the surface of various cell types. Upregulation of ICAM-1 is induced by IL-1, interferon-γ, and TNF-α in response to inflammatory stimuli [60].

Lifitegrast is a medication for the treatment of the symptoms of DED and reduces inflammation of the ocular surface. Lifitegrast mimics ICAM-1, blocks the interaction between cell surface proteins and LFA-1 (which are instrumental in T-cell activation and migration), and interrupts the inflammatory cascade associated with DED [61,62].

Lifitegrast is marketed under the product name Xiidra^®^ and was approved by the U.S. FDA in 2016 for the treatment of DED. In clinical practice it is often used in conjunction with CsA for DED including meibomian gland dysfunction.

The observed adverse drug reactions with Lifitegrast in clinical studies were eye irritation, discomfort, blurred vision, and dysgeusia.

## 4. PACAP as a Candidate Therapy for DED

We recently reported that pituitary adenylate cyclase-activating polypeptide, or PACAP, plays important roles in protecting against DED-like symptoms in mice [63]. Based on that study, PACAP might be clinically useful for the treatment of DED.

### 4.1. Overview of PACAP

PACAP (encoded by the Adcyap1 gene), which exists in 27- or 38-amino-acid isoforms, is first isolated from the extracts of ovine hypothalamus [64,65]. PACAP belongs to the vasoactive intestinal peptide (VIP)/glucagon/secretin family, sharing a 68% amino acid sequence homology with VIP [66]. PACAP27 and PACAP38 have 27 and 38 amino acid residues, respectively, and their activities are similar. In this review, the term PACAP refers to PACAP38 unless otherwise stated. These peptides are produced from the PACAP precursor (pre-pro-PACAP) [67]. PACAP has been markedly well conserved during evolution, with its amino acid sequence being identical in mammals [68,69].

PACAP and VIP share three receptors: the PAC1 receptor (PAC1-R), which has a high affinity for PACAP, the VPAC1 receptor (VPAC1-R), which has a comparable affinity for PACAP and VIP, and the VPAC2 receptor (VPAC2-R), which also displays a comparable affinity for PACAP and VIP. The affinity of PAC1-R for PACAP is more than 1000 times higher than its affinity for VIP [70].

The amino acid sequence of PAC1-R is conserved with a 96.6% homology between mouse and human [71]. The human and mouse VPAC1-R proteins and human and mouse VPAC2-R proteins exhibit 84% and 87% sequence identity, respectively.

PAC1-R is coupled to adenylyl cyclase (AC) and phospholipase C (PLC) through cyclic adenylyl cyclase monophosphate (cAMP); it activates protein kinase A (PKA), which in turn can activate the MAPK pathway. PAC1-R binding can also activate the MAPK pathway independently of AC activation. PLC activation stimulates Ca^2+^ mobilization and protein kinase C (PKC) activation [72].

PACAP and its receptors have diverse biological functions. In the central nervous system (CNS), PACAP acts as a neurotransmitter. In addition, PACAP has been shown to be a neuroprotective effect for cerebral brain ischemia, Parkinson’s disease, traumatic brain injury and spinal injury, oxidative stress, and neurotoxins [72]. In the peripheral nervous system, they function in the control of hormone secretion, vasodilation, and immunosuppression [73].

Moreover, PACAP is known to be involved in the neurite protrusion elongation. PAC1-R is the main pathway in the process of neurite protrusion elongation induced by PACAP. Furthermore, PACAP was found to promote neurite protrusion elongation by promoting the dephosphorylation of collapsing response mediator protein 2 (CRMP2) [74].

### 4.2. Distribution of PACAP and Its Receptors

PACAP is widely distributed in the brain and peripheral organs and tissues in mammals. It is present in the lacrimal gland, choroid, iris, ciliary body, conjunctiva, sclera, cornea, and retroocular arteries of the eye [75,76]. Further distributional analysis at the ultrastructural level by use of electron microscopy showed that PACAP is found in the plasma membrane, rough endoplasmic reticulum, the cytoplasmic matrix of neurons in the inner nuclear layer (INL), in amacrine and horizontal cells, and in the ganglion cell layer (GCL) [77].

PAC1-R is widely detected in the CNS and peripheral organs, including the adrenal glands, testis, anterior pituitary gland, pancreas, and placenta [69]. It is also expressed in the GCL, INL, and amacrine cells and weakly expressed in the IPL and outer nuclear layer (ONL) of the rat retina [77].

VPAC1-R is mainly expressed in the cerebral cortex and the hippocampus, whereas VPAC2-R is predominantly expressed in the hippocampus, thalamus, hypothalamus, the central nucleus of the amygdala, and the pontine nuclei of the brainstem, respectively [78]. In the eye, VPAC1-R and VPAC2-R were barely detectable in the retina, unlike PAC1-R [79].

### 4.3. Tear Fluid Secretion by PACAP

We recently reported that PACAP stimulates tear secretion and plays important roles in protecting against DED symptoms in mice [63]. Over the past few years, PACAP-null (*Adcyap1^–/–^*, PACAP KO) mice have been generated by several laboratories, and their phenotypes have been analyzed. PACAP KO mice are not embryonically lethal and have no apparent abnormalities at a young age. However, PACAP KO mice do exhibit impaired fertility, although the mechanism by which PACAP influences reproduction is only partially understood [80].

During the routine housing of PACAP KO mice in our animal facility, we unexpectedly discovered that some mice displayed eye abnormalities. In these mice, the surface of the eyes appeared white, due to corneal keratinization, and angiogenesis was found to occur in the substantia stroma of the cornea. By fluorescein staining analysis, which is commonly used to visualize the status of the cornea, strong fluorescence suggestive of corneal injury was observed in the central part of the cornea in these mice.

In this analysis, female PACAP KO mice showed a relatively high frequency of corneal keratinization compared to male mice. Moreover, corneal keratinization was more prominent in aged female mice than in males. These results are consistent with the fact that DED correlates with old age and affects females to a greater degree. Because these phenotypes are a common feature of DED symptoms in humans, we thought that a reduction in tear secretion volume might be the cause of the corneal keratinization.

To test the hypothesis, tear secretion levels in PACAP KO mice were measured by Schirmer’s test using cotton thread. A reduction in tear secretion was found in male and female PACAP KO mice aged 10 weeks or older compared with wild-type mice. Based on these observations, we hypothesized that PACAP was associated with altered tear secretion by the lacrimal gland, the major source of tear secretion. To clarify the function of PACAP in tear secretion, PACAP38 solution dissolved in saline was instilled, and the level of tear secretion was again measured by Schirmer’s test. Eye drops containing 0.1 to 10 nM of PACAP38 significantly increased tear secretion from 15 to 45 min after treatment, with levels returning to baseline at 120 min. A PACAP27 eye drop solution also significantly increased tear secretion, whereas VIP (sharing 68% homology of its amino acid sequence with PACAP) did not. Moreover, the continuous administration of PACAP eye drops suppressed corneal keratinization. A similar treatment also suppressed corneal injury and inflammation (unpublished results, manuscript in preparation).

Next, we examined the signaling cascade associated with PACAP-induced tear secretion. Pre-treatment with PACAP6–38 (a PAC1-R antagonist) significantly suppressed PACAP-induced tear secretion, whereas VIP6–28 (a VPAC1-R and VPAC2-R antagonist) did not suppress tear secretion in wild-type mice.

PACAP activates Gs protein signaling via its receptors, such as that involving cAMP production and the phosphorylation of PKA, a cAMP-dependent protein kinase [72]. The level of cAMP in mouse infraorbital lacrimal glands was increased after the administration of PACAP eye drops, and the signal for phosphorylated PKA was also significantly increased. However, pre-treatment with an AC inhibitor suppressed PACAP-induced tear secretion. These results suggest that PACAP-induced tear secretion is mediated by the AC/cAMP/PKA signaling pathway through PAC1-R.

**Figure 1:**
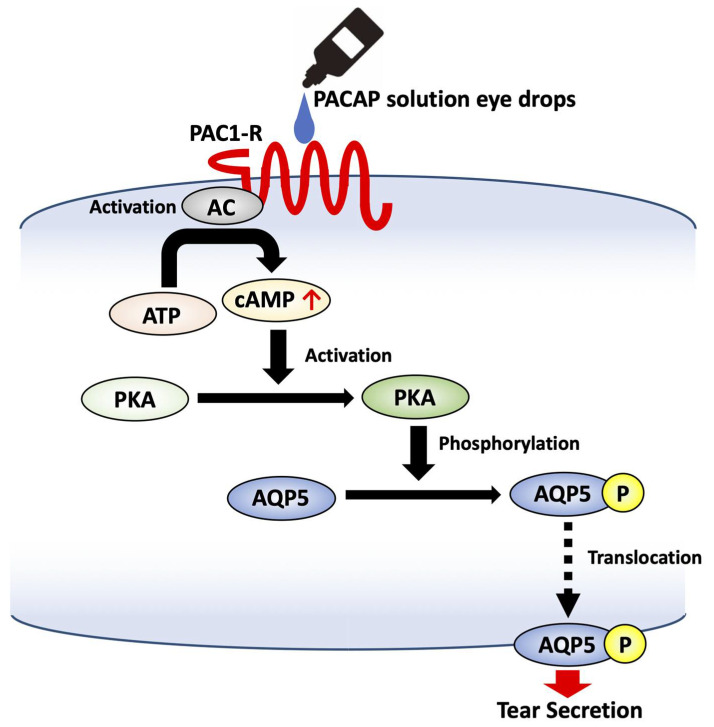
Schematic diagram of signal transduction for PACAP in the lacrimal gland.

Aquaporins (AQPs) are a family of water channel proteins that form pores in the cell membrane, facilitating the transport of water between cells. AQPs are expressed in various tissues and organs, with expression of the AQP4 and AQP5 subtypes being reported in the lacrimal gland [81]. Expression of AQP5 is dramatically decreased in lacrimal acinar cells of patients with Sjögren’s syndrome, a chronic autoimmune disease that affects the body’s moisture-producing capacity, including the function of lacrimal and salivary glands. [82]. The decreased expression of AQP5 levels in Sjögren’s syndrome patients suggests that AQP5 is related to the reduction in tear secretion. It has been reported that activation of the cAMP/PKA cascade can induce the translocation of AQP5 from the cytosol to the apical membrane [83]. In addition, as detected by X-ray crystallography, phosphorylation of AQP5 is required to induce a conformational change for trafficking [84]. PACAP eye drops increased the expression of AQP5 in the membrane and of phosphorylated AQP5 levels in the infraorbital lacrimal glands [63]. On the other hand, AQP5 siRNA treatment of the infraorbital lacrimal gland attenuated PACAP-induced tear secretion. These results suggest that PACAP induces tear secretion via an AC/cAMP/PKA cascade, which in turn stimulates AQP5 translocation from the cytosol to the membrane of lacrimal acinar cells to bring about an increase in water permeability (Figure 1). Our results thus indicate a potentially new function of PACAP as a candidate therapy for DED patients.

Concerning the safety of PACAP, we reported that PACAP eye drops did not cause any adverse reactions in acute to semi-acute phases of dry eye at a concentration of 100 nM, which is a concentration 1000 times higher than the effective dose of PACAP (0.1 nM), or in response to chronic eye drop treatment at a concentration of 0.1 nM for 3 weeks in PACAP KO mice [63]. These data suggest that PACAP acts locally to induce tear secretion without causing toxicity.

It should be noted that there are issues relating to the instability of peptide drugs such as PACAP. In a recent study, the stability of PACAP38 and PACAP27 was measured by LC-MS in four common media (0.9% saline, benzalkonium chloride solution for ophthalmic use, thimerosal solution for ophthalmic use, and water for injection) and in commercially available AT at both room temperature (RT) and 4 °C. After 2 weeks, PACAP38 showed higher stability at 4 °C than at room temperature in common media, especially as more than 90% of the original peptide was measured in 0.9% saline and water for injection at 4 °C. PACAP38 showed higher stability than PACAP 27 in all media at both experimental temperatures [85].

The above findings with PACAP should provide the impetus for further preclinical and clinical studies on the efficacy of PACAP eye drops to treat DED patients.

PACAP induces tear secretion via an AC/cAMP/PKA cascade, which in turn stimulates AQP5 translocation.

## 5. Conclusions

DED has serious deleterious effects on physical and psychological health and quality of life. Important societal costs are attributable to these symptoms in terms of direct costs of care and lost productivity. New therapies are expected to be developed in the coming years, which will significantly increase options for DED patients and result in better control of their condition.

This review summarizes DED medications. Meanwhile, materials to improve the symptoms of DED have been developed.

The polymer including 2-methacryloyloxy ethyl phosphorylcholine (MPC, under the product name Lipidure^®^) is hydrating multifunction agent. A recent study showed that MPC polymer protected corneal cells from desiccations [86]. Furthermore, MPC polymer enhanced the volume of tear fluid in animal models of DED [Nagai 2021]. To date, soft contact lenses containing this MPC have been approved by the Food and Drug Administration (FDA) [87].

Recent research on DED treatment has focused on stimulating tear secretion, mucin secretion, and suppressing inflammation, rather than simply replenishing the ocular surface with water to improve symptoms.

Gabapentin (GBT) is a structural analog of gamma-amino butyric acid that is used as an antiepileptic drug as well as to provide pain relief in glaucomatous patients [88]. GBT also has analgesic and anti-inflammatory properties, so it may be useful as a DED medication [89].

We recently reported that PACAP induces tear secretion, thus demonstrating PACAP as a new candidate medication for DED [63]. While PACAP stimulates lacrimal secretion, mucin secretion has not been clarified. A comparison between PACAP and commonly used DED drugs is shown in Table 1.

Further research needs to be conducted on the clinical applications of PACAP in DED.

## Figures and Tables

**Table 1:** A comparison between PACAP and commonly used DED drugs.

Medications	Water Supplementation. Retention	Anti-inflammation	Suppretion of Injury	Mucin Secretion	Tear Secretion	Product Names
Artificial tears	+ [22]	-	-	-	-	Many products
Hyaluronic acid	+ [90]	-	+ [29]	-	-	Hyalein^®^
Loteprednol Etabonate	-	+ [33]	-	-	-	Lotemax^®^ Loterex^®^
Cyclosporin A	-	+ [39]	+ [40]	-	+ [38]	Restasis^®^
Rebamipide	-	-	-	+ [47,48]	+ [50]	Mucista^®^
Diquafosol	-	+ [55]	+ [55]	+ [55,63]	+ [55]	Diquas^®^
Lifitegrast	-	+ [61,62]	-	-	-	Xiidra^®^
PACAP	-	+ [*]	+ [*]	?	+ [*]	

+: Reported [ref], * Unpublished results, manuscript in preparation. -: Not reported. ?: Unknown.

## Data Availability

Not applicable.
